# A mixed-methods approach to understanding domestic dog health and disease transmission risk in an indigenous reserve in Guyana, South America

**DOI:** 10.1371/journal.pntd.0010469

**Published:** 2022-06-10

**Authors:** Marissa S. Milstein, Christopher A. Shaffer, Phillip Suse, Aron Marawanaru, Daniel A. Heinrich, Peter A. Larsen, Tiffany M. Wolf

**Affiliations:** 1 Department of Veterinary and Biomedical Sciences, College of Veterinary Medicine, University of Minnesota, St. Paul, Minnesota, United States of America; 2 Department of Veterinary Population Medicine, College of Veterinary Medicine, University of Minnesota, St. Paul, Minnesota, United States of America; 3 Department of Anthropology, Grand Valley State University, Allendale, Michigan, United States of America; 4 Masakenari Village, Konashen Indigenous District, Region 9, Guyana; 5 Department of Veterinary Clinical Sciences, College of Veterinary Medicine, University of Minnesota, St. Paul, Minnesota, United States of America; Swiss Tropical and Public Health Institute: Schweizerisches Tropen- und Public Health-Institut, SWITZERLAND

## Abstract

Domestic dogs (*Canis lupus familiaris)* can transmit a variety of pathogens due to their ubiquitousness in urban, rural and natural environments, and their close interactions with wildlife and humans. In this study, we used a mixed-methods approach to assess the role of domestic dogs as potential intermediaries of disease transmission from wildlife to humans among indigenous Waiwai in the Konashen Community Owned Conservation Area, Guyana. To address these objectives we 1) performed physical examinations and collected biological samples to assess Waiwai domestic dog health, and 2) administered questionnaires to characterize the role of dogs in the community and identify potential transmission pathways between wildlife, dogs, and humans. We observed ectoparasites on all dogs (n = 20), including: fleas (100%), ticks (15%), botflies (30%), and jigger flea lesions (*Tunga penetrans)* (80%). Ten percent of dogs were seropositive for *Ehrlichia canis/ewingii*, 10% were positive for *Dirofilaria immitis*, and one dog was seropositive for *Leishmania infantum*. All dogs (n = 20) were seronegative for: canine distemper virus, *Brucella canis*, *Leptospira* serovars, *Trypanosoma cruzi*, *Anaplasma phagocytophilum/platys* and *Borrelia burgdorferi*. Our questionnaire data revealed that the Waiwai remove ectoparasites from their dogs, clean up dog feces, and administer traditional and/or Western medicine to their dogs. White blood cell, strongyle-type ova, and eosinophil counts were lower in dogs that were not frequently used for hunting, dogs that did receive traditional and/or western medicine, and dogs that were frequently kept in elevated dog houses, although differences were not statistically significant. While our results suggest that the Waiwai have developed cultural practices that may promote dog health and/or prevent zoonotic disease transmission, more research is necessary to determine the efficacy of these practices. Our study provides important data on the health of dogs and the potential for disease transmission to humans in a zoonotic hotspot.

## Author summary

Domestic dogs are found throughout the world and interact closely with both wildlife and humans. Dogs can harbor numerous diseases that can be transmitted to other domestic and wildlife species, and human populations. They can serve as bridges, moving diseases between unconnected populations. Additionally, dogs can help in the early detection of wildlife and human diseases. Therefore, understanding what pathogens domestic dogs harbor can inform both wildlife and human health. In this study, we performed physical examinations, collected biological samples, and administered questionnaires to assess dog health and understand the role of dogs as potential bridges of disease transmission from wildlife to humans among indigenous Waiwai in the Konashen Community Owned Conservation Area, Guyana. On physical exam, we observed ectoparasites on all dogs, however, few pathogens were detected with diagnostic screening. Our questionnaire data revealed that the Waiwai engage in husbandry practices that may promote Waiwai dog health.

## Introduction

Domestic dogs (*Canis lupus familiaris)* can play important roles in the transmission of a variety of pathogens. These include parasitic (*Leishmania spp*., *Neospora caninum*, *Dirofilaria immitis*, *Echinococcus spp*.) [1–7], viral (rabies, distemper, parvovirus) [8–12] and bacterial (*Leptospira* serovars, *Brucella canis*, *Rickettsia rickettsii)* [13–15] pathogens. The ubiquitousness of domestic dogs in urban, rural and natural environments, as well as their close interactions with wildlife and humans, make dogs uniquely suited to serve as reservoirs, sentinel species and/or bridge hosts for these and other pathogens. In Serengeti National Park, a canine distemper virus outbreak in lions originated from a high-density domestic dog population [9,16,17] and dogs were implicated as a maintenance reservoir for rabies, which caused population declines of wild dogs and lions in the ecosystem [8,18,19]. In South America, domestic dogs are a main reservoir for visceral leishmania (*Leishmania infantum)*, a zoonotic protozoan and major public health threat [20].

Domestic dogs can be sentinel species (i.e. a species that indicates the presence of a disease [21,22]) and bridge hosts (i.e. a host that facilitates pathogen transmission between two unconnected host populations [23]) for human or wildlife diseases. Dogs are unique in these roles as they i) have similar pathogen exposure patterns to their human counterparts, ii) are often susceptible to human and wildlife infections, iii) freely interface and move between wildlife and human populations iv) scavenge for wildlife and v) are relatively easy to capture and handle for biological sampling [12,24,25]. Indoor dogs, which likely have similar exposure patterns to their owners, were found to be a sensitive indicator of human West Nile virus infection risk [26]. Dogs are also highly susceptible to *Trypanasoma cruzi*, the zoonotic protozoan of Chagas disease. The primary vector of *T*. *cruzi*, *Triatoma infestans*, preferentially feeds on domestic dogs, making them an ideal indicator species for Chagas disease surveillance [27]. Further, the movement of free-ranging dogs between wildlife and human populations facilitates the translocation of, for example, ectoparasites [28], many of which harbor zoonotic pathogens, including: *Bartonella*, *Rickettsia*, and *Dipylidium* [28–30]. Finally, because dogs are relatively easier to capture compared to free-ranging wildlife species, they can be vital in sentinel surveillance when wildlife disease data are lacking. Anthrax surveillance in dogs has been useful in the detection and mapping of anthrax in areas where there are limited data on outbreaks [25].

Further, the role of domestic dogs in infectious disease transmission may be particularly important in communities that use dogs for hunting. Among the indigenous populations of the Guianas in South America, dogs have been essential components of subsistence hunting practices for hundreds of years [31]. Researchers have long recognized the close cultural and livelihood connections that many Amazonian peoples have with their dogs. The Waiwai, an indigenous community located in Southern Guyana, are particularly well-known for their skilled hunting dogs, which were highly valued culturally and economically [32–35]. According to ethnographers who lived with the Waiwai in the middle of the 20th century, dogs were given personal names, adorned with feather collars, and washed daily [33]. The physical appearance (particularly strength and perceived health) and the behavior of dogs, including where they roamed and how aggressive they were, was viewed by the Waiwai as a reflection of their owners [33,34]. A dog that wandered too much or was poorly behaved caused others in the community to censure the owner [33,34].

In addition to their importance in aiding Waiwai hunters in hunting large game (e.g. peccaries and tapir), Waiwai hunting dogs were prized trade objects, and were traded throughout Northern Amazonia [31]. Related to the high value placed on them, the Waiwai were reported to engage in several practices intended to promote strength and health in their dogs. Until the 1960s, Waiwai dogs were kept on elevated shelves or hammocks within the common house, with the Waiwai perceiving that this was necessary to prevent infection from ectoparasites, especially jigger fleas (*Tunga penetrans*) [33]. Dogs were collared and tied loosely to the shelf walls, preventing them from coming to the ground until they were untied. They were reported to be meticulously housetrained, waiting until they were led away from the communal house to defecate or urinate [33]. Dogs were administered a variety of topical and internal medicines derived from plants and casts were placed to repair broken bones [33]. According to Guppy [32]:

Even more than the English the Wai-Wais deserve to be famous as dog-lovers. Their dogs are, collectively, the world’s most pampered—nothing is too good for them. When puppies are born they are washed at once in a special infusion of roots to make them strong. If their mother dies or runs dry they may be suckled by a woman. They spend most of their adult days reclining, in the ease of Oriental potentates, on their shelves, or even in their own hammocks, above the ground out of reach of jigger-fleas. They are fed on the pick of the kill; and they are washed two or three times a day, partly for cleanliness, but mostly to prevent them suffering from the heat.

The extent to which these practices continue and their efficacy for preventing parasitic infection, is unclear. Our team’s preliminary ethnographic data indicate that Waiwai dogs may be exposed to a variety of parasites and may contribute to pathogen transmission from wildlife to humans [36]. We observed Waiwai dogs frequently scavenging cooked and uncooked organs and tissues from wildlife harvested for food by the Waiwai and regularly interacting with wildlife during hunting and farming activities. We also found that dogs were important for improving Waiwai hunting success and are in regular close contact with people throughout the village [36]. During meetings with the community in 2018, many Waiwai were concerned that their dogs were not as healthy or strong as they had been in the past, and expressed a keen interest in obtaining a baseline assessment of the general health of Waiwai village dogs.

In this study, we used a mixed-methods approach to assess the general health of Waiwai domestic dogs and their role as potential intermediaries of disease transmission from wildlife to humans. Our specific objectives were to characterize the overall health of Waiwai domestic dogs, screen for potentially zoonotic pathogens in dogs, and determine cultural practices that may promote dog health and/or minimize the transmission of zoonotic pathogens. To address these objectives we 1) performed physical examinations and used diagnostic screening to assess Waiwai domestic dog health, and 2) administered questionnaires to characterize the role of domestic dogs in the community and identify potential transmission pathways between wildlife, domestic dogs, and humans.

## Materials and methods

### Ethics statement

We followed the ethical standards of the Institutional Review Boards of the University of Minnesota and Grand Valley State University and with the 1964 Helsinki declaration and its later amendments or comparable ethical standards. All domestic dog procedures were approved by the Institutional Animal Care and Use Committee at the University of Minnesota (Protocol ID: 1904-36989A). Research was approved by the Environmental Protection Agency and the Ministry of Indigenous People’s Affairs in Guyana and the Waiwai Village Council.

### Study site

**Fig 1:**
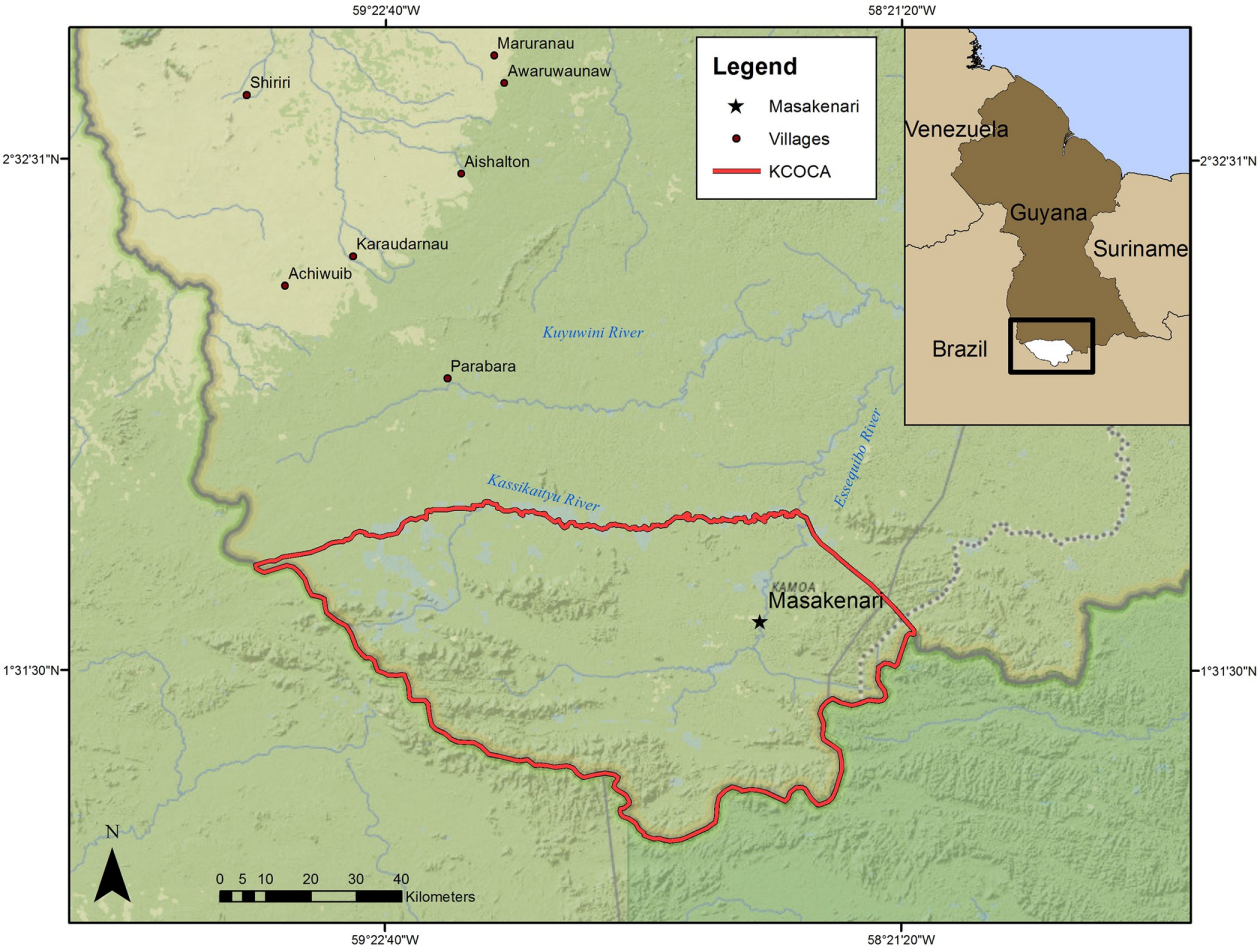
Map of the the Konashen Community Owned Conservation Area (KCOCA) and the nearest villages. Service Layer Credits: Sources: National Geographic, Esri, DeLorme, HERE, UNEP-WCMC, USGS, NASA, ESA, METI, NRCAN, GEBCO, NOAA, iPC.

The Konashen Community Owned Conservation Area (KCOCA) is a 625,000 ha indigenous reserve in Southern Guyana, South America (Fig 1). The KCOCA is owned and managed by indigenous Waiwai, contains high biodiversity, and protects a portion of one of the world’s largest areas of contiguous rainforests. Approximately 225 Waiwai live in the KCOCA, concentrated in the village of Masakenari [36–38]. Within the KCOCA, no other human or domestic dog population resides outside of Masakenari. The Waiwai practice a traditional Amazonian subsistence strategy of swidden cassava horticulture and supplement their diet with fish and wild meat [37].

Our team has been collaborating with the Waiwai of Masakenari since 2013 on a multidisciplinary project focusing on sustainable resource use and health [36–40]. In order to prioritize Waiwai research interests and goals, this project has involved extensive community-based, ethnographic research and community engagement, with team members living in the village during several months each year [36,39,40]. During this time, the research team observed that all dogs were owned by Waiwai families who provisioned food (i.e. dried cassava, scraps of meat and fish) and shelter (in the form of traditional dog houses). Waiwai dogs were also observed scavenging for food throughout the village and although generally not allowed inside the home, dogs would occasionally enter kitchens to scavenge for food. Dogs were allowed to freely roam, but they primarily stayed within the village and close to their households. Dogs accompanied their owners while farming and hunting, and sometimes crossed the river and entered the forest edge alone or with other dogs [36].

As dogs are frequently traded and roam freely around the relatively spatially dispersed village of Masakenari, obtaining an exact count of the number of dogs was not possible. Therefore, to estimate the approximate total number of dogs in the village, CAS asked the head of each of the 45 households in the village how many dogs they had during ethnographic research in 2018 (the year before this study). This provided an estimate of 50–60 village dogs.

### Physical examinations and sample collection

To assess domestic dog health, we collected data on 20 Waiwai dogs during an eight-day study period from July 4, 2019 to July 12, 2019. Because Masakenari is a relatively spatially broad village and dogs in different parts of the village may have different environmental exposures, our sampling procedure involved dividing the village into four spatial areas and randomly selecting four households from each of these areas. In each area, we sampled one dog from three households and two dogs from one household, for a total of 20 dogs. Although the remoteness of the study site and the time constraints associated with using liquid nitrogen for sample preservation prevented us from sampling more than 20 dogs, our sample represented approximately 35% of the dogs in the village.

We performed physical examinations on each dog to assess: heart and respiratory rate, temperature, weight (estimated by eye based on the following ordinal scale: 2-4kg, 5-7kg, 8-10kg, 11-13kg, 14-16kg), body condition (Scale: 1–9; following guidelines established by the World Small Animal Veterinary Association [WSAVA]) and muscle mass (Scale: 1—severe muscle wasting, 2—mild to moderate muscle wasting, 3—normal muscle; modified scoring guidelines from WSAVA), ectoparasite load, and skin and coat condition. We collected up to 20 milliliters of blood, which we aliquoted into sterile, red-top tubes for serum separation and K-EDTA purple-top whole blood tubes. We initially screened whole blood on site (see details below) and created dried blood smears on glass slides for transport at room temperature to the University of Minnesota College of Veterinary Medicine (UMN-CVM) Veterinary Medical Center (VMC) for further examination. We stored serum samples in liquid nitrogen in the field and in a -80C freezer once back at the UMN-CVM. We collected freshly voided fecal samples from 16 of the 20 dogs to screen for gastrointestinal parasites. We mixed two to three grams of feces with 10 milliliters of 10% buffered formalin and stored fecal samples at room temperature.

### Diagnostic screening

In a temporary, field-based laboratory, we measured packed cell volume (PCV), total protein (TP), total white blood cell (WBC) counts, and screened for heartworm and tick-borne diseases using whole blood. We manually calculated WBC counts using a Whi-pette Test Kit (Exotic Animal Solutions LLC, United States) according to manufacturer’s instructions. We screened dogs for heartworm (*Dirofilaria immitis*) antigens, and tick-borne (*Borrelia burgdorferi*, *Ehrlichia canis/ewingii*, *Anaplasma phagocytophilum/platys*) antibodies using 4DX SNAP Tests (IDEXX, United States). At the UMN-CVM VMC Clinical Pathology Laboratory, we stained the dried blood films with automated Wright-Giemsa stain and estimated differential WBC counts, red blood cell morphology and screened for hemoparasites. We used reference intervals established by the UMN-CVM VMC Clinical Pathology Laboratory from healthy, client and/or employee owned pet dogs presenting to the UMN-CVM VMC between the ages of 1–10 years without historical systemic illness and normal physical exam findings. Of note, these intervals are based on total WBC counts completed by an automated hematology analyzer and not via a manual methodology; however, manual differentials are part of the reference interval generation.

To screen for zoonotic pathogens harbored by domestic dogs, we submitted serum samples for: *Leishmania* and *T*. *cruzi* (indirect fluorescent antibody tests) to Texas A&M Veterinary Medical Diagnostic Laboratory, *B*. *canis* (tube agglutination) and *L*. *interrogans* serovars *bratislava*, *canicola*, *grippotyphosa*, *hardjo*, *icterohemorrhagica*, *pomona* (microscopic agglutination test) to the UMN Veterinary Diagnostic Laboratory, and canine distemper virus (antibody serum-neutralization) to Colorado State Veterinary Diagnostic Laboratory. Lastly, we submitted fecal samples to the UMN Veterinary Diagnostic Laboratory for quantitative egg counts using the Modified Wisconsin Sugar Floatation Technique.

### Questionnaires and risk analysis

We verbally administered questionnaires to each of the owners of the same 20 dogs for whom we collected physical and diagnostic data. Questionnaires consisted of 37 closed-ended questions about dog care, uses, disposition, and interactions with wildlife (see S1 Table). In cases where multiple dogs from the same household were sampled, each questionnaire applied to a specific dog within that household (i.e. whether that specific dog was kept in a doghouse rather than whether that household had a doghouse). Even within the same household, each dog had an individual as its owner, thus questionnaire data originates from unique, individual owners and represents unique, individual study animals. Questionnaire questions were developed during ethnographic research in 2018 [36], and discussions with key informants were used to assure questions were phrased in ways that would minimize misinterpretation among respondents [41]. Questionnaires were administered face-to-face during extended visits to each household, as is culturally appropriate in Waiwai society. All dog owners selected for the questionnaires readily agreed to participate in this study. We summarized questionnaire data with descriptive statistics using SPSS (Version 26, IBM, United States).

Since the Waiwai have been observed engaging in husbandry practices that were suggested to support the health and vitality of their dogs [32–34], we used diagnostic and questionnaire data from this study to test those relationships. We hypothesized that specific husbandry practices may be associated with differences in white blood cell, eosinophil, and strongyle-type ova counts. We hypothesized that, 1) dogs kept on elevated shelves would have lower WBC, strongyle-type ova, and eosinophil counts; 2) dogs that were regularly administered traditional medicines would have lower WBC, strongyle-type ova, and eosinophil counts; and 3) dogs that were frequently used for hunting (hunting more than once per month) would have significantly different WBC, strongyle-type ova, and eosinophil counts than non-hunting dogs. We selected the indicators WBC, strongyle-type ova, and eosinophil counts based on the recognition that the WBC count is often used as a non-specific screening test for general inflammation in domestic dogs [42,43], and eosinophilia, or increased eosinophils in the blood, can be associated with either internal or external parasites or hypersensitivity reactions, particularly, flea-bite dermatitis [43–45]. Although increases in WBC and eosinophil count are non-specific and may be insensitive for some underlying pathologies, they may also indicate inflammation/infection that we have not tested for in specific pathogen tests. Further, strongyle-type ova, which are associated with canine hookworm infection, are a zoonotic helminth with a soil reservoir, commonly found in Amazonian communities [46,47]. Thus, as the practice of keeping dogs in elevated dog houses has been suggested to alleviate the impacts of ectoparasites [33] or reduce exposure to soil-associated helminths [32,33], evidence of these relationships may be demonstrated as lower WBC, strongyle-type ova, and eosinophil counts. As some traditional medicinal practices have been associated with health benefits [48–50], we might also see evidence of this as lower WBC, strongyle-type ova, and eosinophil counts among dogs regularly administered traditional medicines. We defined traditional medicines as those derived from local plants or animals that our Waiwai informants reported as being used indigenously to treat or prevent ailments prior to the availability of Western medicines, and defined dogs receiving traditional medicine as those that had been treated with these medicines within the past year. Because some dogs in our sample received Western medicines, we also tested the relationships between the use of Western medicine (and Western medicine combined with traditional medicines) and these response variables. Finally, Waiwai hunting dogs have previously been reported to receive more care from their owners and appear larger and healthier than non-hunting dogs [33–35]; thus, evidence of better health might be reflected by lower WBC, strongyle-type ova, and eosinophil counts. However, hunting dogs, both directly from their interactions with prey, and indirectly through their increased time spent in the forest [51–54], may have exposure to a greater diversity of parasites [55] or experience more trauma related to hunting activities, which may be reflected by higher WBC, strongyle-type ova, and eosinophil counts [56]. Previous studies at other sites have shown higher diversity and prevalence of parasites among hunting dogs compared to non-hunting but free roaming dogs [52,53,56,57]. In addition, hunting dogs with higher parasitic loads have been shown to exhibit higher WBC and eosinophil counts [51,55,58]. Therefore, we hypothesized that hunting dogs would differ significantly from non-hunting dogs in WBC, strongyle-type ova, and eosinophil counts, but did not predict the direction of this relationship.

We tested for associations using Mann-Whitney U tests for non-normally distributed data using SPSS. We set the alpha level for all statistical tests a priori at ≤0.05. Standard errors were estimated for means and we calculated 95% Wilson exact confidence intervals for percentages in Epitools [59]. Wilson exact confidence intervals are appropriate when p is extreme [60]; in this study many proportions were close to 0.

## Results

### Dog health screening

Twenty Waiwai dogs were sampled across 16 households. Eighty percent (n = 16) of the dogs were male and 20% (n = 4) female. Mean age was 2.8 years (SD ±2.5 years) and mean body temperature was 102.1 Fahrenheit (F) (SD ±1.1F; RI: 100.2–103.8F). The mode weight range was 5-7kg (Range: 2-13kg), median body condition score was 3.5 (Range: 2.5–5.0), median muscle condition 3.0 (Range: 2.0–3.0) (Table 1).

**Table 1:** Selected physical exam findings from Waiwai domestic dogs (n = 20 dogs) and results from differential white blood cell counts, packed cell volume (PCV), and total protein (TP). Reference intervals for blood cell parameters from the UMN-CVM VMC Clinical Pathology Laboratory were included for comparison.

Parameter	Units	Mean	s.d.	Median	Minimum Range	Maximum Range	RI^a^
Age	Years	2.8	2.5	2.0	0.4	9.0	n/a
BCS	1–9	n/a	n/a	3.5	2.5	5.0	Scale: 1–9
MCS	1–3	n/a	n/a	3.0	2.0	3.0	Scale: 1–3
Temperature	Fahrenheit	102.1	1.1	102.4	99.9	104.2	100.2–103.8
Heart Rate	beats/minute	110	24	104	68	160	70–120
Respiratory Rate^b^	beats/minute	n/a	n/a	n/a	12	Pant	18–34
WBCs	cells/uL	17,265	5,465	15,648	10,395	29,315	3,880–14,570
Neutrophils	cells/uL	9,440	3,156	9,278	4,574	16,524	2,100–11,200
Lymphocytes	cells/uL	3,652	1,423	3,269	1,615	7,704	780–3,360
Eosinophils	cells/uL	3,270	3,271	2,173	756	14,658	0–1,200
Monocytes	cells/uL	507	321	461	0	1,106	0–1,200
Basophils	cells/uL	409	349	346	0	1,153	0–130
PCV	%	39.5	6.0	39.5	30.0	51.0	37.5–60.3
TP	g/dL	6.4	1.0	6.5	5.3	10.0	5.8–7.2

^a^Reference intervals established from healthy, owned pet dogs, presenting to the UMN-CVM VMC between the ages of 1–10 years.

^b^Respiratory Rate: 5 of 20 dogs were panting at time of physical exam and therefore summary statistics could not be calculated.

**Fig 2:**
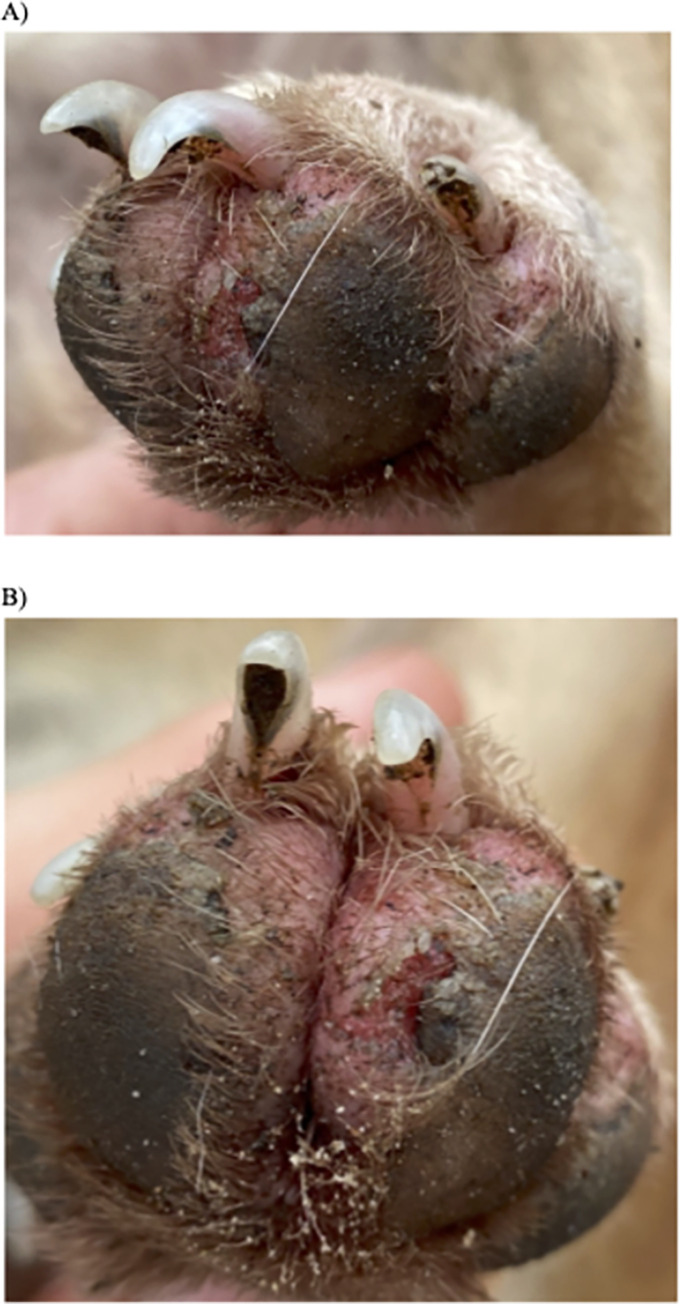
Jigger flea (*Tunga penetrans)* lesions on Waiwai dog paw pads. A) Female jigger fleas (*Tunga penetrans*) penetrate the skin and lay eggs around the toes and soles of feet. B) Dogs will remove jigger fleas with their teeth, creating lesions in the paw pads.

On physical examination, one dog was observed to have vampire bat (Desmodontinae) bites on the ears and nape of the neck. Eighty percent (n = 16) of dogs had evidence of past and/or current jigger flea lesions on their paw pads (Fig 2A and 2B). Other flea species were observed on the fur of all dogs. Fifteen percent (n = 3) of dogs presented with 1–10 ticks, no ticks were appreciated on the remaining 17 dogs. Thirty percent (n = 6) were observed with past and/or current bot fly (*Dermatobia hominis*) lesions. Thirty percent (n = 6) were observed with ocular abnormalities including: increased corneal opacity, corneal lesions, epiphora, and enophthalmos.

We found that mean counts of WBCs, lymphocytes, eosinophils and basophils were above the reference interval (Table 1). Mean WBC count was 17,265 cells/ul (SD ±5,465; RI: 3,880–14,570 cells/ul), mean lymphocyte count was 3,652 cells/ul (SD ±1,423; RI: 780–3,360 cells/ul), mean eosinophil count was 3,270 cells/ul (SD ±3,271; RI: 0–1,200 cells/ul), and mean basophil count was 409 cells/ul (SD ± 49; RI: 0–130). Mean segmented neutrophils, monocytes, PCV, and TP were all within the reference interval (Table 1). No hemoparasites or significant changes in red blood cell morphology were observed. Reactive lymphocytes were appreciated on all blood films

Ten percent of dogs (n = 2) were positive for *D*. *immitis*. Ten percent of dogs (n = 2) had antibodies against *E*. *canis/ewingii*. The dogs that tested positive for *D*. *immitis* and that had antibodies against *E*. *canis/ewingii* were unique individuals (n = 4 total dogs tested positive on 4DX). All dogs (n = 20) lacked antibodies against *A*. *phagocytophilum/platys* and *B*. *burgdorferi*. One dog had antibodies against *L*. *infantum* at a titer level of 1:64 (weak positive). All dogs (n = 20) lacked antibodies against *B*. *canis*, canine distemper virus, *L*. *interrogans*, and *T*. *cruzi*. (Table 2).

**Table 2:** Results from 4DX SNAP tests and serological tests from Waiwai domestic dogs (n = 20).

	Test Type	N	Percent Positive	95% CI
*Dirofilaria immitis* (Heartworm)	4DX–Antigen	2	10	2.8–30.1
*Ehrlichia canis/ E*. *ewingii*	4DX–Antibody	2	10	2.8–30.1
*Anaplasma phagocytophilum/A*. *platys*	4DX–Antibody	0	0	0.0–16.1
*Borrelia burgdorferi* (Lyme disease)	4DX–Antibody	0	0	0.0–16.1
*Leishmania infantum*	Indirect Fluorescent Test–Antibody	1	5	0.9–23.6
*Brucella canis*	Tube Agglutination–Antibody	0	0	0.0–16.1
*Canine distemper virus*	Serum Neutralization–Antibody	0	0	0.0–16.1
*Leptospira* serovars	Microscopic Agglutination Test–Antibody	0	0	0.0–16.1
*Trypanasoma cruzi*	Indirect Fluorescent Test–Antibody	0	0	0.0–16.1

We collected fecal samples from a total of 16 dogs, which were examined for gastrointestinal parasites. Eighty-eight percent of dogs (n = 14) were observed with strongyle-type ova, mean strongyle-type ova was 52 eggs per gram (SD ±77), and three dogs had greater than 100 eggs per gram. Twenty-five percent (n = 4) were observed with *Capillaria* species, mean *Capillaria* species was 1.1 eggs per gram (SD ±3).

### Questionnaires and risk analysis

The 16 households where sampling took place had a total of 47 dogs. Seventy-five percent (n = 15) of respondents classified their dog sampled in the study as a hunting dog, although only 35% (n = 7) said that their dogs went hunting more than once per month. Ninety-five percent (n = 19) of owners stated that their dog consumed raw meat and 65% (n = 13) of dogs consumed primate entrails (ie. organs and tissues). Eighty-five percent (n = 17) of owners stated that their dogs eat bacaba (*Oenocarpus bacaba*). Ten percent (n = 2) stated that their dog had been bitten by primates and 70% (n = 14) of dogs had been bitten by bats in the past year. None of the owners reported that their dogs exhibited signs of rabies infection. All owners stated that they have observed their dogs cross the river and 25% (n = 5) stated that their dogs travel into the forest without being accompanied by their owners. Fifteen percent (n = 3) stated that their dogs brought back wildlife to the household. Ninety percent (n = 18) stated that they remove dog fecal material from around their homes and 80% (n = 16) stated that their dogs will enter their homes. Fifty-five percent (n = 11) of respondents stated that they tie their dog in an elevated dog house but none of the owners kept their dogs on dog shelves (in the home). Twenty percent (n = 4) stated that they administer only traditional medicine to their dogs, 10% (n = 2) only administer western medicine (when available) and 25% (n = 5) provide both. Western medicines included topical antibacterial and antifungals, and occasionally antihelminthic medicines. Traditional medicines included chile peppers to improve hunting ability; crushed stems from the barbasco plant (*Lonchocarpus urucu*), the bitter juice extracted from the cassava plant (*Manihot esculenta*) during food processing that contains cyanogenic glucosides [61], and leaves of *Alocasia* spp. for fleas and ticks; and *Himatanthus sucuuba* for diarrhea (Tables 3 and S1).

**Table 3:** Selected structured interview questions and results (n = 20 dog owners).

Question^a^		N	Percent Responses	95% CI
Is your dog a hunting dog?				
	Yes	15	75	53.1–88.1
	No	5	25	11.2–46.9
How frequently does the dog hunt?				
	>1/week	1	5	0.9–23.6
	1/week	6	30	14.6–51.9
	1/month	7	35	18.1–56.7
	<1/month	1	5	0.9–23.6
	Never	5	25	11.2–46.9
Does the dog consume raw meat?				
	Yes	19	95	76.4–99.1
	No	1	5	0.9–23.6
Does the dog consume primate entrails?				
	Yes	13	65	43.3–81.9
	No	7	35	18.1–56.7
Is the dog kept on a dog shelf in your home?				
	Yes	0	0	0.0–16.1
	No	20	100	83.9–100
Is the dog kept in a doghouse?				
	Yes	11	55	34.2–74.2
	No	9	45	25.8–65.8
What type of medicine does the dog receive?				
	Only western	2	10	2.79–30.1
	Only traditional	4	20	8.1–41.6
	Both	5	25	11.2–46.9
	None	9	45	25.8–65.8
What type of traditional medicine does the dog receive? (owners could respond with more than one answer)				
	*Lonchocarpus urucu*	9	45	25.8–65.8
	*Himatanthus sucuuba*	8	40	21.9–61.3
	*Alocasia* spp.	7	35	18.1–56.7
	*Capsicum* spp.	9	45	25.8–65.8
	Other	5	25	11.2–46.9
Does the dog consume *O*. *bacaba*?				
	Yes	17	85	64.0–94.8
	No	3	15	5.2–36.0
What stage of O. bacaba processing does the dog consume? (owners could respond with more than one answer)				
	Fruit	7	35	18.1–56.7
	Mash	17	85	64.0–94.8
	Waste	1	5	0.89–23.6
	Porridge	1	5	0.89–23.6
	Processed Drink	4	20	8.1–41.6
Has the dog been bitten by wildlife?				
	Yes	2	10	2.8–30.1
	No	18	90	69.9–97.2
Has the dog been bitten by vampire bats?				
	Yes	14	70	48.1–85.5
	No	6	30	14.6–51.9
Has the dog ever exhibited any of the following behaviors [characteristic of rabies]: increased responsiveness to auditory/ visual stimuli, irritability, biting inanimate objects, pupillary dilation, inappetance, difficulty swallowing/drinking water, excess salivation				
	Yes	0	0	0.0–16.1
	No	20	100	83.9–100
Does the dog bring back wildlife to the home?				
	Yes	3	15	5.2–36.0
	No	17	85	64.0–94.8
Does the dog travel into the forest without you or another person?				
	Yes	5	25	11.2–46.9
	No	15	75	53.1–88.1
Do you clean the dog’s feces from your household?				
	Yes	18	90	69.9–97.2
	No	2	10	2.8–30.1
Do you remove ticks from this dog?				
	Yes	18	90	69.9–97.2
	No	2	10	2.8–30.1
Do you remove jigger fleas from this dog?				
	Yes	18	90	69.9–97.2
	No	2	10	2.8–30.1
Does the dog enter your home?				
	Yes	16	80	58.4–91.9
	No	4	20	8.1–41.6

^a^See S1 Table for the full list of interview questions.

Dogs regularly used for hunting had higher mean WBC, strongyle-type ova, and eosinophil counts than non-hunting dogs, although these results were not significant (Table 4). Dogs that received medicine had lower mean WBC, strongyle-type ova, and eosinophil counts than those that did not receive any type of medicine and these differences were more pronounced when analyzing dogs that specifically received traditional medicines but were not significant. Dogs that were regularly kept in dog houses also had lower mean strongyle-type ova, WBC, and eosinophil counts. While WBC (p = 0.06) and eosinophil counts (p = 0.13) approached significance, these results were also not significant at the alpha level of 0.05.

**Table 4:** Comparison of white blood cell, strongyle ova, and eosinophil counts across four independent variables predicted to be associated with Waiwai dog health.

Variable	N	White Blood Cell Count				Strongyle Ova Count				Eosinophils Count			
	20	Mean	s.e.	Mann- Whitney U	p- value	Mean	s.e.	Mann-Whitney U	p- value	Mean	s.e.	Mann-Whitney U	p- value
Hunting dog				54.00	0.53			61.00	0.24			66.00	0.12
Y	7	18087	1915			53.29	31.03			3407.8	616.9		
N	13	16821	1613			33.77	19.16			3195.4	1095.1		
Any Medicine				38.00	0.41			55.00	0.71			33.00	0.21
Y	11	16205	1575			29.27	13.93			3170.2	1230.8		
N	9	18559	1926			54.44	32.26			3391.4	710.9		
Traditional medicine^a^				31.00	0.18			43.00	0.66			36.00	0.33
Y	9	15541	1803			19.33	14.67			3159.8	1500.0		
N	11	18675	1609			58.00	26.36			3404.1	610.2		
Doghouse				24.00	0.06			51.00	0.94			29.00	0.13
Y	11	14880	992			36.55	22.32			2236.6	489.9		
N	9	20179	2109			45.56	24.78			4532.4	1447.1		

^a^ Traditional medicine was defined as those derived from local plants or animals that Waiwai informants reported as being used indigenously to treat or prevent ailments prior to the availability of Western medicines, and we defined dogs receiving traditional medicine as those that had been treated with these medicines within the past year.

## Discussion

In this study, we used traditional veterinary diagnostics and quantitative surveys to assess the health of Waiwai domestic dogs and their potential role in pathogen transmission between wildlife and humans. We observed ectoparasites, including: fleas, ticks, and botflies on all dogs (n = 20) during physical examination. All dogs were seronegative on diagnostic screening for *Leptospira* serovars, *Brucella canis*, and *Trypanosoma cruzi*, with one dog a weak positive for *Leishmania infantum* antibodies. Only two dogs tested positive for *Dirofilaria immitis* (heartworm) and another two dogs had antibodies against *E*. *canis/ewingii*. Overall, we found WBC parameters that exceeded those of our reference dog population, including: total WBCs, eosinophils, basophils and lymphocytes. The majority of dogs had strongyle-type ova on fecal parasitology. Our questionnaire data revealed that the Waiwai engage in behaviors intended to promote dog health, such as removing ectoparasites from their dogs, cleaning up dog feces around their homes, and keeping dogs in traditional, elevated dog houses.

On physical examination, we observed ticks on 15% (n = 3) of dogs, and 10% (n = 2) of dogs had antibodies against *E*. *canis/ewingii*. The small number of dogs with appreciable ticks on physical examination is consistent with the questionnaire data, which revealed that all dog owners actively removed ticks from their dogs. The removal of ticks from dogs may limit the transmission of tick-borne diseases within the Waiwai domestic dog population and/or from dogs to the Waiwai community. *E*. *canis*, the causative agent of canine monocytic ehrlichiosis, is a common, globally distributed tick-borne disease that is highly prevalent in tropical (eg. South American and Caribbean) climates [62]. Transmitted by the brown dog tick (*Rhipociephalus sanguineus*), *E*. *canis* primarily infects dogs; however, there have been accounts of *E*. *canis* infections in other mammalian species, including humans [63]. Domestic dogs can serve as both the reservoir host for *E*. *canis* and the maintenance host for *R*. *sanguineus* [64]. Given that 10% of dogs were seropositive for *E*. *canis* and that many tick-borne diseases screened in this study are zoonotic (ie. *A*. *phagocytophilum*, *B*. *burgdorferi*, *E*. *canis/ewingii*), continued surveillance of tick-borne diseases through the use of rapid screening tests like 4DX SNAP tests are important for the prevention of human tick-borne diseases.

Despite the lack of heavy tick burdens among Waiwai dogs, we observed jigger fleas/sand flea lesions on the majority of Waiwai dogs. Tungiasis is a neglected tropical disease caused by a female jigger flea. Female jigger fleas penetrate the skin and lay eggs around the toes and soles of feet, resulting in acute inflammation. Erythema, edema, swelling and itching was appreciable on all dogs with jigger flea infection. Tungiasis is a zoonotic disease that affects both animals and humans, with domestic animals, such as dogs, as the main animal reservoir [65]. Animal reservoirs are important in transmission, as the fleas will reach maturity and reproduce in animal hosts while humans often remove the fleas before reproduction occurs.

**Fig 3:**
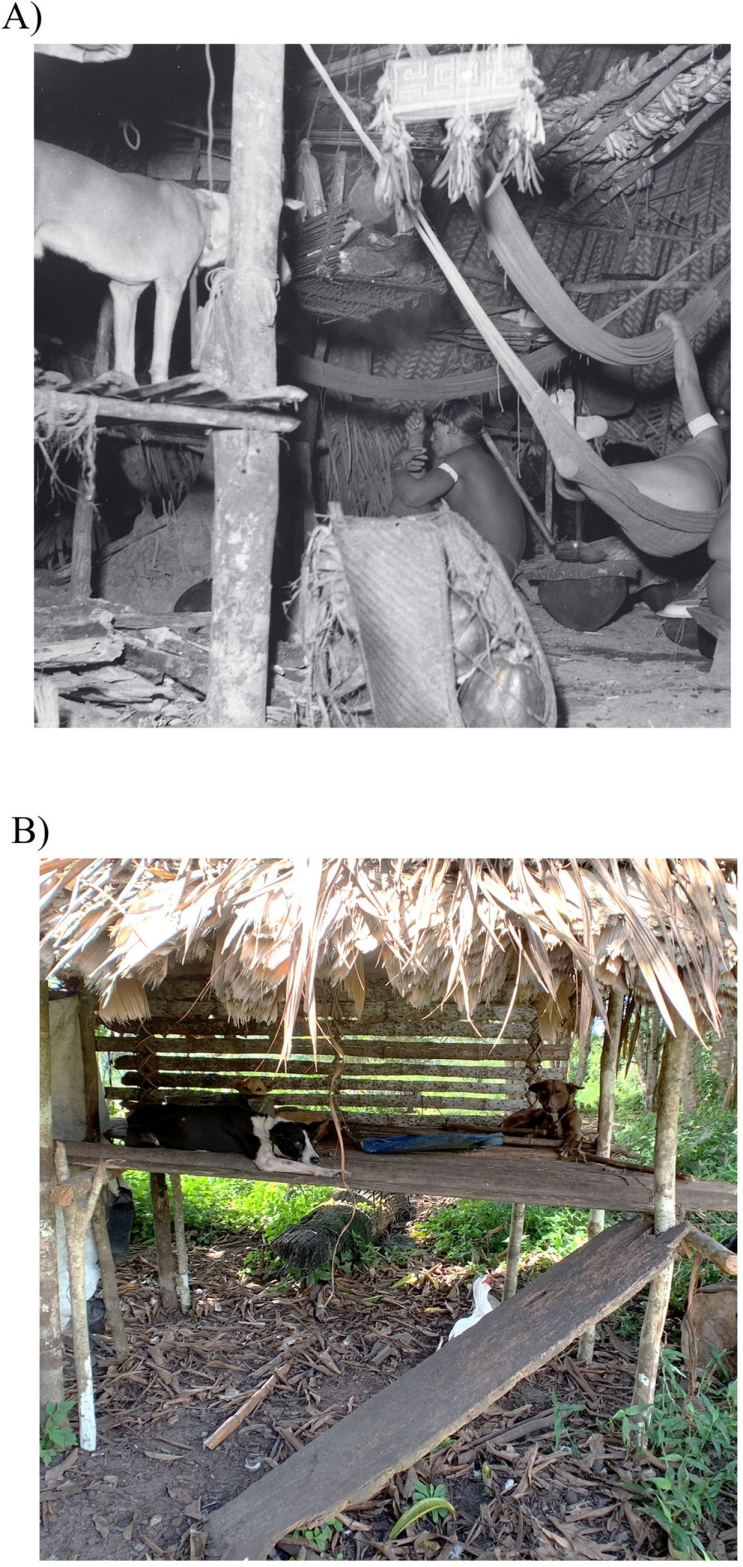
Comparison of Waiwai dog shelves used in the past and current Waiwai dog houses. A) View of the interior of a communal Waiwai house in Konashen, Guyana circa 1955, showing a dog leashed on a dog shelf on the left. Scanned black and white negative. Interior with hammocks, three people and a dog. Negative of slide 2. Object no: ARC/GUP/004/003 Horniman Museum and Gardens. B) Dog house in Masakenari Village, Guyana in 2019.

Our questionnaire data revealed that the Waiwai engage in several practices designed to mitigate tungiasis in their dogs. For example, most owners reported that they frequently removed jigger flea egg sacs from their dogs’ paws. In addition, dog houses were placed on stilts, with respondents identifying jigger fleas and other parasites as the primary reason for them being elevated. Until the 1960s, Waiwai dogs were kept on elevated shelves or hammocks within the common house (Fig 3A), primarily to prevent infection with ectoparasites [33]. Dogs were collared and tied loosely to the shelf walls, preventing them from coming to the ground until they were untied. While none of the dogs in our study were kept on dog shelves, this practice appears to be partially retained in the now-external doghouses (Fig 3B). However, given the still high frequency of jigger flea infection in sampled dogs, the extent to which these practices are effective at reducing tungiasis in both dogs and humans in the community is unclear.

One possibility is that the shift from keeping dogs permanently tied on shelves to keeping them less regularly in elevated dog houses has increased the prevalence of jigger fleas in dogs. While there have been no previous studies of tungiasis in Waiwai dogs, ethnographers of the Waiwai in the middle of the 20th century described their dogs as relatively free of parasites and directly attributed this to the dogs only being on the ground when on leashed walks, carried, or hunting in the forest [32,33]. In contrast, Waiwai dogs in this study were free to roam the village when not tied in their dog houses, and even dogs that were regularly put in dog houses would spend much of their time outside on the ground. Unfortunately, without data on the frequency of tungiasis in dogs prior to the shift away from dog shelves, it is not possible to test whether jigger flea prevalence has increased as a result of this shift. In many parts of the tropical world, including South America, tungiasis causes considerable morbidity and even mortality in dogs and people [66–69]. Therefore, an increased prevalence of jigger fleas would represent a major public health concern for the community.

In the current study, all dogs were seronegative for *T*. *cruzi*. This finding is surprising given that the vast majority of Waiwai owners stated that dog houses had thatched roofs (a common habitat for triatomine insects) and that dogs consume bacaba (i.e. Turu palm, *O*. *bacaba*) and raw meat. In addition to vector borne transmission by triatomine insects (*Triatoma*, *Panstrongylus*, *Rhodnius*), food borne transmission in human populations is increasing [70]. Around 70% of acute Chagas disease in the past decade was associated with foodborne transmission, most commonly from drinks made with the wild acai (*Euterpe oleracea)* and bacaba [71]. Bacaba is processed into a drink that is both culturally and nutritionally important for the Waiwai [33,72] and other indigenous Amazonians [73]. *T*. *cruzi* has also been shown to infect a wide variety of mammalian species, including several of the most important prey species for the Waiwai, such as primates and large rodents [74]. Although little is known about food borne transmission of Chagas disease in domestic dogs, previous studies have shown that dogs exhibit pathogen susceptibility and exposure routes (vector-borne and food-borne) similar to humans [75,76]. Chagas disease is a major public health threat throughout Central and South America [76,77]. While we detected no evidence of previous exposure to *T*. *cruzi* among the Waiwai dogs, this finding warrants further investigation, especially given our small sample size.

Only a single Waiwai dog had antibodies against *L*. *infantum*, whose titer level was detectable at 1:64 (routine dilution series for this assay is 1:64 through 1:2048), which alone is not diagnostic of active *Leishmania* infection [78]. Dogs can be chronically infected with *L*. *infantum* without an appreciable antibody titer. We observed no characteristic ulcerative skin lesions on any of the physical examinations performed on the Waiwai dogs, and no amastigotes were noted on peripheral blood films. Indeed, the presence of amastigotes on blood film is rare [79–81], making a diagnosis even more challenging. Interestingly, the dog that was seropositive for *L*. *infantum* was acquired from Waiwai communities in Northwest Brazil and not raised in the Waiwai village of Masakenari. Therefore, it is also possible this dog was infected with *L*. *infantum* prior to having been brought to the area. Since dogs are a major reservoir for *L*. *infantum* infection in humans, dogs brought from outside of the community may potentially serve as a pathway for zoonotic transmission of *L*. *infantum* into the community.

Overall, we observed evidence of leukocytosis with corresponding lymphocytosis, eosinophilia and basophilia. In addition, while the results were not significant, the mean total WBC and eosinophil counts among dogs that were administered some form of medicinal treatment or preventative and/or that were kept in elevated dog houses were lower than dogs that were not cared for in those ways. Eosinophilia and basophilia are commonly associated with inflammatory responses to parasitism [44]. The reference intervals used in this study were generated from a representative, healthy dog population specific to the UMN-CVM VMC. To the best of the authors’ knowledge, there are no reference intervals for free-ranging dogs in this specific region. Therefore, these reference intervals may not appropriately reflect the baseline health of the Waiwai dog population, as these differences likely reflect differences in antigenic exposure and lack of routine anti-parasitic prophylaxis. Nevertheless, these general white blood cell parameters provide important baseline information about the health of Waiwai dogs and their antigenic exposures.

Our questionnaire data revealed that dogs were frequently bitten by vampire bats and we observed vampire bat bites (on the ears and nape of neck) in one dog on physical examination. Vampire bats are reservoirs for many zoonotic viruses [82,83], particularly rabies [84], and commonly feed on the blood of domestic species [85,86]. Even with increased recognition of vampire bats as a major public health threat in rural Amazonia [87], they remain understudied in South America [83]. Despite the lack of data on the local risk of rabies transmission from vampire bats in the Waiwai village, vampire bats are the main animal reservoir for rabies in Guyana, Suriname, and French Guiana [88]. Although no respondents in our study reported that any of their dogs had ever exhibited signs consistent with rabies, the relatively dense dog population coupled with the frequency of vampire bat bites, is a potential risk for future rabies outbreaks. Therefore, better characterizing pathogen transmission between bats, dogs and humans is a priority area for future research in this community.

We found that 14 of the 16 fecal samples analyzed showed evidence of strongyle-type ova, with some samples exhibiting greater than 100 eggs per gram of feces. While we were unable to identify the eggs at the species or genus level, the presence of these eggs in canine feces is likely consistent with canine hookworms, a zoonotic helminth that can cause cutaneous larval migrans in humans. Reichert et al. [47] identified several risk factors for hookworm associated cutaneous larval migrans in Manus, Brazil including: presence of free-roaming dogs, lack of veterinary care and preventative anti-helminthics, presence of animal feces, walking barefoot (especially children), and tropical environmental conditions (temperature and precipitation) [47]. Almost all Waiwai respondents in this study indicated that they clean dog feces from around their homes and we directly observed that Waiwai yards were clean of feces. Diligent removal of dog feces may decrease the transmission of canine zoonotic gastrointestinal parasites, specifically, canine hookworms to humans. However, the rest of the risk factors identified by Reichert et al. [47] are similar to what was observed in the Waiwai village. Therefore, we suggest that veterinary interventions should focus on the administration of anti-parasitic dewormers to target canine hookworms, such as fenbendazole and pyrantel, to minimize the potential of zoonotic transmission.

A high percentage of respondents administered either traditional (24%) or Western medicine (10%), or both (25%), to their dogs on a regular basis. While the efficacy of some of these treatments is unestablished, we identified several traditional or “bush” medicines that have demonstrated antiparasitic or anthelmintic properties. For example, several respondents reported topical application of crushed stems from the barbasco plant (*Lonchocarpus urucu*) to treat severe flea and tick infections. *L*. *urucu* contains rotenone, a widely used insecticide [89]. Respondents also reported using *Himatanthus sucuuba* to treat dogs that are too skinny or who are suffering from diarrhea. *H*. *sucuuba* is commonly used as a medicinal plant throughout South America, having anti-inflammatory [48], anti-bacterial [50], and even anti-leishmanial properties [49]. While our results were not statistically significant, dogs administered traditional medicine had lower fecal strongyle ova counts as compared to dogs that were not treated. Given the limited statistical power from our relatively small sample size, further research is necessary to ascertain the extent to which these traditional medicines are efficacious against zoonotic canine helminths.

Ninety-five percent of owners stated that their dogs consume wildlife entrails and our previous research has shown that despite how the Waiwai discarded entrails, dogs would almost always find and consume them [36]. As the Waiwai are highly reliant on wild meat, particularly that of primates and rodents, the consumption of entrails likely represents the primary risk for dogs acquiring pathogens from wildlife.

We found that dogs kept in houses and those administered traditional medicines had lower WBC counts, and these results neared significance at the p < 0.05 level. However, the efficacy of these practices for decreasing parasitic infections is unclear. The Waiwai have been known for the quality of their hunting dogs and their close relationship with these dogs since their first interactions with Europeans in the mid 19th century [33]. Several ethnographers have reported that the Waiwai engaged in practices specifically designed to promote the health of their dogs, including keeping them on elevated dog shelves inside the house, administering a variety of topical and internal medicines derived from plants, carefully controlling their movements, diligently cleaning feces, and even splinting broken bones [32,33]. Indeed, domestic dogs are highly valued culturally and economically by many indigenous populations throughout Amazonia [90,91]. Several of these groups have been reported to use a variety of ethnoveterinary medicines for their dogs [92–94], including some of the medicines that we documented in this study. To our knowledge, however, the use of elevated doghouses is found only among the Waiwai and the speakers of a few closely related Carib languages in Suriname, French Guiana, and Northern Brazil (e.g. the Trio, Hixkaryana, Wayana) [32–34,95,96]. Like the Waiwai, these groups are highly reliant on hunting dogs (some of which are obtained through trade with the Waiwai), and appear to have used hunting dogs prior to first contact with Europeans [34,95–97]. Therefore, an interesting area for future research is the extent to which these groups may have developed traditional practices for promoting dog health.

Our study had several limitations. In regard to the serological diagnostic tests used in the study (ie. *T*. *cruzi* IFA, Distemper SN, and *B*. *canis* TAT), we were unable to find information on test performance (eg. sensitivity, specificity, positive and negative predictive value). This limitation is important to note given that these tests are often considered the reference standard for diagnostic screening (ie. Distemper SN [98], *T*. *cruzi* IFA [99,100]), are commonly used in clinical and population screenings (ie. *B*. *canis* TAT [98]), and the lack of data on test performance pose serious challenges in test interpretation and population health. Previous studies have found the *Leishmania* IFA highly sensitive and specific (sensitivity = 96%, specificity = 98%) [101], with the potential for false positives if the animal is exposed to *Trypanosoma spp*. [98], as well as the SNAP 4DX Test (Heartworm: sensitivity = 99.0%, specificity = 99.3%; *Anaplasma*: sensitivity = 90.3%, specificity = 94.3%; *Ehrlichia*: sensitivity = 97.1%, specificity = 95.3%; *B*. *burgdorferi*: sensitivity = 94.1%, specificity 96.2%) [102]. Additionally, although no dogs were seropositive on Leptospirosis MAT tests, false negatives are common, especially early in the course of the disease. For example, a single titer measurement has been estimated to have a sensitivity of 50% (with specificity of 100%) [103]. Therefore, we cannot conclude that leptospirosis is not a health problem in this population on a single negative titer alone.

Despite these limitations, our mixed-methods study provides important data on the health of domestic dogs in a zoonotic hotspot [104]. Such research is essential for better understanding shared human-dog health and mitigating zoonotic disease transmission throughout the tropical world. Our diagnostic screening showed little evidence of zoonotic pathogens circulating in Waiwai domestic dogs and our questionnaire data revealed several cultural practices of the Waiwai intended to promote the health of their dogs. Given that dogs have been demonstrated to be important sentinels and/or bridge hosts for human pathogens [21–24], more research is needed to identify what pathogens are circulating in this region, outside of pathogen-specific serological tests. Employing unbiased next generation sequencing, particularly RNA-based transcriptome research, is an exciting tool for surveillance and discovery, where a priori knowledge of pathogen targets is not needed [105]. Our future work aims at utilizing this approach to identify zoonotic pathogens of domestic dogs as a way to better understand their roles in infectious disease transmission and sentinel surveillance in the region.

## Supporting information

S1 Table Waiwai Dog Health Questionnaire. Closed-ended questions were asked verbally to Waiwai about dog care, uses, disposition, and interactions with wildlife.
(PDF)
