# Supplementary material for: A mixed-methods approach to understanding domestic dog health and disease transmission risk in an indigenous reserve in Guyana, South America
Source: PLoS Negl Trop Dis. 2022 Jun 10;16(6):e0010469. doi: 10.1371/journal.pntd.0010469 (PMC9223617; doi:10.1371/journal.pntd.0010469)
Supplement: S1 Table — Closed-ended questions were asked verbally to Waiwai about dog care, uses, disposition, and interactions with wildlife. (PDF) [file pntd.0010469.s001.pdf]

Supplemental Table 1. Questionnaire questions and responses for 20 respondents in the Konashen Community Owned Conservation Area.

**Question**		**N**	**Percent Responses**
Is this dog male or female?			
	Male	16	80
	Female	4	20
How old is this dog?			
	< 1 year	2	10
	1 – 1.9 years	5	25
	2 – 2.9 years	5	25
	3 – 3.9 years	3	15
	4 – 4.9 years	1	5
	> 5 years	4	20
Where was your dog born?			
	Masakenari (Guyana)	16	80
	Parabara (Guyana)	2	10
	Lethem (Guyana)	1	5
	Mapuera (Brazil)	1	5
Do you clean your dog’s feces from around your home?			
	Yes	18	90
	No	2	10
What foods do you feed your dog?			
	Cassava	20	100
	Scraps from hunted animals	20	100
	Free to scavenge	20	100
	Other	6	30
	None	0	0
Does your dog eat raw meat?			
	Yes	19	95
	No	1	5
Do you cook meat for your dog?			
	Yes	20	100
	No	0	0
Does your dog eat the entrails of primates?			
	Yes	13	65
	No	7	35
Is your dog a hunting dog?			
	Yes	15	75
	No	5	25
How frequently does the dog hunt?			
	>1/week	1	5
	1/week	6	30

	1/month	7	35
	<1/month	1	5
	Never	5	25
Is your dog a good hunting dog?			
	Yes	8	40
	No	7	35
What does your dog eat during hunts?			
	Cassava	15	75
	Scraps from hunted animals	15	75
	Free to scavenge	15	75
Does your dog accompany you to your farm?			
	Yes	19	95
	No	1	5
Does your dog cross the creek on their own?			
	Yes	20	100
	No	0	0
Does your dog go into the forest without you or another person?			
	Yes	5	25
	No	15	75
Is your dog free to roam the village on their own?			
	Yes	19	95
	No	1	5
Is the dog kept on a dog shelf in your home?			
	Yes	0	0
	No	20	100
Is the dog kept in a doghouse?			
	Yes	11	55
	No	9	45
What type of roof does the doghouse have?			
	Thatch	9	45
	Zinc	2	10
How frequently do you tie your dog in the doghouse?			
	One day per week	4	20
	Two to three days per week	4	20
	Everyday	3	15
What type of medicine does the dog receive?			
	Only western	2	10
	Only traditional	4	20
	Both	5	25
	None	9	45

What type of traditional medicine does the dog receive? (owners could respond with more than one answer)			
	Lonchocarpus urucu	9	45
	Himatanthus sucuuba	8	40
	Alocasia spp.	7	35
	Capsicum spp.	9	45
	Other	5	25
What are the traditional medicines used for?			
	Flea/tick (*Lonchocarpus urucu*, *Alocasia* spp., *Manihot esculenta*)	9	45
	Improve hunting ability/decrease laziness (*Capsicum* spp.)	9	45
	Diarrhea (*Himatanthus sucuuba*)	8	40
What type of Western medicine does the dog receive? (Owners could respond with more than one answer)			
	Topical antibacterial	5	25
	Topical antifungal	4	20
	Antihelminthic	2	10
Does the dog consume *O. bacaba*?			
	Yes	17	85
	No	3	15
What stage of *O. bacaba* processing does the dog consume? (owners could respond with more than one answer)			
	Fruit	7	35
	Mash	17	85
	Waste	1	5
	Porridge	1	5
	Processed Drink	4	20
Has the dog been bitten by wildlife in the past year?			
	Yes	2	10
	No	18	90
Has the dog brought home wildlife in the past year?			
	Yes	3	15
	No	17	85

Has the dog been bitten by vampire bats in the past year?			
	Yes	14	70
	No	6	30
Has the dog ever exhibited any of the following behaviors [characteristic of rabies]: increased responsiveness to auditory/ visual stimuli, irritability, biting inanimate objects, pupillary dilation, inappetance, difficulty swallowing/drinking water, excess salivation			
	Yes	0	0
	No	20	100
Has the dog ever exhibited other strange or abnormal behaviors?			
	Yes	16	80
	No	4	20
What strange behaviors has the dog exhibited?			
	Laziness	7	35
	Weakness and lethargy	6	30
	“Sad”	4	20
	Coughing	3	15
	Not eating	3	15
	Other	3	15
Has the dog ever bitten any person?			
	Yes	1	5
	No	19	95
Has the dog fought with other dogs in the past year?			
	Yes	20	100
	No	0	0
Do you remove ticks from this dog?			
	Yes	18	90
	No	2	10
Do you remove jigger fleas from this dog?			
	Yes	18	90
	No	2	10
Does the dog enter your home?			
	Yes	16	80
	No	4	20
